# Assessment of antibiotic storage practices, knowledge, and awareness related to antibiotic uses and antibiotic resistance among household members in post-conflict areas of Pakistan: Bi-central study

**DOI:** 10.3389/fmed.2022.962657

**Published:** 2022-09-08

**Authors:** Faiz Ullah Khan, Tauqeer Hussain Mallhi, Qasim Khan, Farman Ullah Khan, Khezar Hayat, Yusra Habib Khan, Tawseef Ahmad, Yu Fang

**Affiliations:** ^1^Department of Pharmacy Administration and Clinical Pharmacy, School of Pharmacy, Xi'an Jiaotong University, Xi'an, China; ^2^Center for Drug Safety and Policy Research, Xi'an Jiaotong University, Xi'an, China; ^3^Shaanxi Center for Health Reform and Development Research, Xi'an, China; ^4^Research Institute for Drug Safety and Monitoring, Institute of Pharmaceutical Science and Technology, Western China Science & Technology Innovation Harbor, Xi'an, China; ^5^Department of Clinical Pharmacy, College of Pharmacy, Jouf University, Sakaka, Saudi Arabia; ^6^Department of Pharmacy, COMSATS University Islamabad, Abbottabad, Pakistan; ^7^Institute of Pharmaceutical Sciences, The University of Veterinary and Animal Sciences, Lahore, Pakistan; ^8^Department of Clinical Pharmacy, Faculty of Pharmaceutical Sciences, Prince of Songkla University, Songkhla, Thailand

**Keywords:** antibiotics storage, antibiotic resistance, households, post-conflict areas, Pakistan

## Abstract

**Background:**

The storage of antimicrobials at home is frequently in-practice in various developing countries, resulting an irrational use, antibiotic resistance, and toxicities. This condition may worsen more in conflict zones where health facilities are limited. This study aimed to determine the storage and use of leftover antibiotics among households (HHs) along with knowledge and awareness about antibiotics and antibiotic resistance (ABR).

**Methods:**

A descriptive cross-sectional study design was employed. Members of HHs were invited to participate in the survey while using a convenient sampling technique. The data were obtained using a validated questionnaire and analyzed through SPSS.

**Results:**

A total of 96 HHs were randomly selected from two districts (*n* = 50, *n* = 46), with most of the participants being men between the ages of 18 and 28 (*n* = 45, 46.9%) years. The majority of HHs (*n* = 32, 33.3%) had six to eight total family members, with one to two chronic diseases (*n* = 63, 65.6%), individual families (*n* = 60, 62.5%), and with (*n* = 35, 36.5%) LRTIs (lower respiratory tract infections). The HHs were aware of the word “antibiotic” (*n* = 59, 61.5%) and gave correct replies to amoxicillin as an antibiotic (*n* = 42, 43.8%); on the other hand, HHs also thought of paracetamol as an antibiotic (*n* = 45, 47.9%). They identified the most common brands of antibiotics easily, and a majority of them (*n* = 69, 71.9%) had never heard of ABR before and had lower levels of awareness. The most stored antibiotic at home (n=38, 39.6%) was azithromycin (J01FA10). In addition, they had multiple needless (1–2, *n* = 62, 64.6%; 3–4, *n* = 29, 30.2%) and antibiotics in their houses. Age had a strong association (*p* = 0.017, H = 12) affected the mean knowledge scores related to antibiotic use. Association of education levels (*p* = 0.001, H = 52.8) and occupation (*p* = 0.04, H = 10) with proper antibiotics use were found to be significant. However, family members with more than one chronic illness (*p* = 0.09, H = 0.8) showed a significant relationship with their awareness of antibiotics.

**Conclusion:**

Participants generally stored various antibiotics of different classes in their homes. Lack of knowledge related to the appropriate usage of antibiotics, use of leftover antibiotics, and awareness related to ABR were unknown to the participants.

## Introduction

A majority of households (HHs) around the world keep medicines on hand for a variety of reasons, including the treatment of both short-term and long-term illnesses, as well as for emergencies. If the directions for storage are not followed, drug stability may be impacted, which could result in ineffective therapy (1, 2). The therapeutic effectiveness of antimicrobial medicines is dependent on their capacity to selectively target invasive bacteria without damaging host cells, yet their manufacturing and stability are key challenges (3). However, the risk of accidental poisoning by unintentional users such as children has also been associated with drug sharing and reusing (2). Conversely, antibiotics can also have negative effects. The most frequent negative side effects related to antibiotic use include allergic responses, ototoxicity, hepatotoxicity, phototoxicity, and nephrotoxicity (4). Regardless, stockpiling of medicines in HHs has been on the rise, given the impact of violence and conflict in many parts of society, including the health sector. In war zones, antibiotic storage in HHs is particularly prevalent (5).

Conflict or war is described as “a condition of open, typically long-term combat between two parties or opponents.” Conflicts have significantly harmed health systems across the world, resulting in delayed access to care, overcrowding, and shortages of medical supplies (6). As a result, the storage of antibiotics at home has grown with an aim to utilize any leftover antibiotics in the future. Unsupervised antibiotic storage leads to antibiotic abuse, which is a result of unneeded antibiotic storage in HHs in conflict zones worldwide (7, 8). In combat zones, self-medication with antibiotics is one type of abuse, which refers to treating self-diagnosed diseases without contacting a physician or under the supervision of a doctor and pharmacist (8, 9). Antibiotics are often purchased over-the-counter across the developed and developing world, with around half of all antibiotics received without a prescription (10, 11). According to microbiological data analysis by Médecins Sans Frontières (MSF), third-generation cephalosporin and carbapenem resistance is 86.2% and 4.3% respectively among Enterobacteriaceae isolates; MRSA (methicillin-resistant *Staphylococcus aureus*) is found in 60.5% patients, and resistance types and rates are similar in patients from Yemen, Syria, and Iraq (12). The National Institute of Health in Islamabad reported that conflict is believed to have led to widespread drug-resistant typhoid fever, which could affect over 2,000 individuals in Pakistan within 6 months. Only one oral antibiotic, azithromycin, was shown to be sensitive (13). Intravenous medications, for example, are costly and impractical in low-income nations. Antimicrobial self-medication has been linked to leftover antibiotics (14, 15). This means that keeping antibiotics in the home increases the likelihood of irrational antibiotic usage. Antibiotics are among the most routinely kept drugs in homes (16, 17). Studies conducted in the United Kingdom and Australia have shown that 19–47% of HHs have stored antibiotics at home including remnant, standby, and current antibiotics (18, 19). Antimicrobial/ABR is a significant public health issue that affects both developed and developing countries. Moreover, the incidence of ABR has risen in recent years, and this, along with the scarcity of antibiotics in the pipeline, has resulted in an ABR crisis. Available evidence indicates that 62% of antibiotics purchased in community pharmacies do not have a prescription (20). Furthermore, self-medication with antibiotics is common, ranging from 19 to 82% (14, 15, 21), and this is usually associated with inappropriate antibiotic use (15, 21). Globally, resistance patterns have been seen among the members of Enterobacteriaceae, *Mycobacterium tuberculosis, Staphylococcus aureus, Streptococcus pneumoniae*, and *Pseudomonas aeruginosa*. Various monitoring systems have been established to check for variations in how susceptible bacteria are to antimicrobial medications. Estimating the worldwide incidence of AMR/ABR will be greatly aided by the availability of updated epidemiological data on frequently encountered diseases exhibiting resistance to antimicrobial agents (22). ABR, therefore, has emerged as one of the main issues facing developed and developing countries that require immediate action to stop the misuse of antibiotics.

Swat, a district in the Khyber Pakhtunkhwa region of Pakistan, has witnessed extensive armed conflict since 2007 (23). During the tumultuous conflict between militants and the army in Pakistan in 2009, more than two million people from the valley became internally displaced persons (IDPs) (24). These IDPs were asked to return in July 2009 after the government proclaimed the region peaceful, while the area's ongoing military presence and sporadic clashes between security forces and militants continue to cause societal unrest (25). Currently, the situation in the Swat valley is peaceful, and in the past decade, several infrastructural projects have been initiated in the health sector. However, more work is still needed, especially in rural areas. The objective of this study is to evaluate HH storage practices, uses, knowledge, and awareness about antibiotics and ABR in the post-conflict area of Swat and Shangla in Pakistan.

## Methods

### Study design and location

From November 2020 to April 2021, a bi-center cross-sectional interview-based survey was conducted in the rural parts of two post-conflict districts namely Swat and Shangla in Pakistan. Swat, formally known as the “Yousufzai State of Swat” (1849–1969), is a rural district in Malakand Division Khyber Pakhtunkhwa, Pakistan, with a population of 2.3 million people. About 86% of the total population lives in rural areas. District Shangla was once a part of Swat state but was carved out in 1995 as a separate district and adjunct to the Swat district having a population of 0.7 million (26). The current study is part of the main trial which contributes baseline data to the registered protocol (ChiCTR2000040453) and is a scheduled phase of the research project. The results that have been presented are following the EATSA protocol and serve as a foundation for the beginning of the main trial (27).

### Study population

This study employed a door-to-door survey method, which is an excellent strategy for gathering meaningful measures of health. In the vicinity of both district rural regions, the HHs were prioritized for data collection. Permanent residents of the area or residents who have lived in the area for the past 5 years and do not have a disability were eligible to answer questions and respond to the research teams. Participants under the age of 18, as well as HHs with no male members and those who refused to consent, were all excluded from the study. Following their visits to each HH, the data collection teams (DCTs) received preliminary training to ensure that the norms and local traditions are considered. For a total of 2 weeks, DCTs received prior training daily throughout their working days.

### Validity and reliability

The face and content validity of the questionnaires were evaluated with the help of specialists in the field. The study tool was piloted with a group of 15 HHs to determine its internal consistency. Visits to both research sites were part of the pilot project, which were utilized to validate the questionnaire. This part of the study provided feedback to the research team, confirming that they had a good understanding of the questions (Supplementary File 2).

The internal consistency of the amended questionnaire was calculated using Cronbach alpha on 22 HHs. The Cronbach alpha result (overall = 0.7; each, 0.69) was adequate and acceptable, indicating that the questionnaire was internally consistent.

### Study instruments

Medical records were utilized as data sources, and all the information was self-reported by the participants. The questionnaire requested details on demographic factors (gender, age, location, occupation, education level, family status, and average monthly income), as well as respondents' awareness of antibiotics, ABR, and antibiotic storage procedures at their homes. Each question prompted replies in a variety of ways, including yes/no/don't know, closed-ended questions with numerous answers, and open alternatives. The questionnaire was developed using relevant information from prior studies on the subject (19, 28–32).

The final questionnaire had three sections. Section I sought demographic details and had a total of 10 questions. Section II had questions related to knowledge (antibiotics identification=4, antibiotics use = 6, and antibiotic resistance = 6) of antibiotics and 16 statements to which the respondents were asked to respond. Two false and two true (Amoxicillin is an antibiotic/Paracetamol is an antibiotic) statements were also included in the identification of antibiotics. Three false questions were asked about the use of antibiotics (antibiotics are often needed for cold and flu), and six questions were related to ABR (misuse of antibiotics can lead to ABR) with the correct approach. A total of eight statements were also employed for the assessment of overall HHs' knowledge of antibiotics use, ABR, and infectious diseases. The responses to these statements were evaluated (Likert scale). Section III included 15 statements/questions related to the HH storage of antibiotics and factors related to storage conditions.

### Translation

The questionnaire was translated by two native translators through the forward and backward translation methods. All data was gathered using a structured paper questionnaire with questions written in the local and national languages (Pashto/Urdu). Forward and reverse translation procedures were undertaken by experts due to the questionnaire's cultural adaption.

### Sampling technique and sample size

A convenient sampling technique was adopted to collect data. We intended to recruit as many HHs as feasible over the course of the study to increase the sample's prospective representativeness. As a logical consequence, we did not determine the minimum sample size in advance (33). As a result, ninety-six HHs (50 in Swat and 46 in Shangla) was determined as the quota needs to represent a mix of geography, pharmacy type (independent and chain community pharmacies), and family size in the HH (large, medium, and small; combined or/ individual).

### Data collection

A significant amount of group orientation was provided to the DCTs, covering areas like guidelines for conducting the interview and HHs survey, advice on proper attire to respect the locale's cultural norms, and other specifications. The DCTs were coached by researchers who were familiar with the clinical situations and the rural community environment. Before administering the survey, the researchers and DCTs visited 22 residences (Swat = 12 and Shangla = 10) in both districts jointly to create familiarity and confidence.

### Quality control procedures and data management

Several efforts were made to achieve maximum standardization and preserve consistency in the data gathering. All data were assigned a special code of identification. The research team members entered the data and cross-checked it. All files were stored, and the confidentiality of the data was assured.

### Statistical analysis

Statistical Package for the Social Sciences (SPSS version 21) was used for data entry and data compilation. Additional test statistics were employed to compare categorical variables, and descriptive statistical analyses were provided as frequencies (percentages). If p>0.05, differences were judged statistically significant. Due to the non-normal data distribution, continuous data were presented as median and interquartile ranges (IQRs). The comparison of continuous data across various samples was conducted through Kruskal–Wallis, and Mann–Whitney tests, where appropriate. Antibiotic use, identification, and ABR median scores (which measure associated characteristics) were also calculated and compared with demographic data such as age, gender, occupation, education level, the total number of families, average income, and family setup. The median scores and testing statistics scores for the types of infectious diseases and total chronic diseases patients present per HH were also tested and tabulated.

## Results

### Demographic characteristics

A total of 96 HHs from two districts (District I: Swat = 50, District II: Shangla = 46) participated voluntarily, resulting in an 88.8% response rate response rate was observed. Considering the cultural values in the study locations, only male members of the HHs took part in the study with the mean age group 18–28 (*n* = 45, 46.9%) years. The majority of its members had primary education (*n* = 28, 29.2%), were self-employed (*n* = 32, 33.3%), and (*n* = 29, 30.2%) with an average monthly income of PKR 21000–30000. Most of the families had six to eight members (*n* = 32, 33.3%), had family members with one or two chronic diseases (*n* = 63, 65.6%), were individual families (n=60, 62.5%), and had prevalence of LRTIs (*n* = 35, 36.5%). A small (*n* = 14,14.6%) number of healthcare professionals were present at home (Table 1).

**Table 1:** Demographic characteristics.

**Demographic variables**	***n* (%)**
Location of the participants*	
District-I	50 (52.1)
District-II	46 (47.9)
**Age of the participants (years)**	
18–28	45 (46.9)
29–38	27 (28.1)
39–48	14 (14.6)
49–58	6 (6.3)
>60	4 (4.2)
**Education level of the participants**	
Primary education	28 (29.2)
Secondary education	18 (18.8)
Higher secondary education	15 (15.6)
Graduation	13 (13.5)
Postgraduation	4 (4.2)
No-formal education	18 (18.8)
**Occupation of the participants**	
Government employee	19 (19.8)
Private employee	23 (24)
Self-business	32 (33.3)
Daily wager	14 (14.6)
Other	8 (8.3)
**Family set-up of the participants**	
Individual	60 (62.5)
Joint family	35 (36.5)
Extended family	1 (1)
**Total number of a family (Male and Female)**	
2–4	4 (4.2)
4–6	27 (28.1)
6–8	32 (33.3)
10–12	21 (21.9)
>12	12 (12.5)
**Average monthly income of the participants (PKR)**	
1,000–20,000	9 (9.4)
21,000–30,000	29 (30.2)
31,000–40,000	21 (21.9)
41,000–50,000	9 (9.4)
>50,000	28 (29.2)
**Total number of patients with chronic diseases**	
0	33 (34.4)
1–2	63 (65.6)
**Presence of the infectious/ other diseases**	
Lower respiratory tract infections (LRTIs)	35 (36.5)
Upper respiratory tract infections (URTIs)	11 (11.5)
Other infectious disease (TB)	13 (13.5)
Chronic illness (Hypertension; Diabetes mellitus)	29 (30.2)
Not any disease (NAD)	8 (8.3)
**Presence of healthcare professionals at home**	
Yes	14 (14.6)
No	82 (85.4)

*Study Locations (District-I=Swat; District-II=Shangla).

### Antibiotic identification, knowledge, and ABR

The overall score for knowledge of antibiotic was relatively good and nearly half of the participants provided correct answers to the statements asked as presented in Table 2. The term antibiotics and knowledge related to antibiotics identification was about 57.3% (*n* = 55). But less than half of the respondents could correctly identify amoxicillin as an antibiotic. Additionally, a majority (61.5%) provided incorrect responses to the correct use of antibiotics and believed that ABs were efficacious in flu and cold. About half of the participants answered the side effects statements and reported that antibiotics kill useful bacteria and ABs can cause (67.7%) of allergic reactions. However, they were unaware of ABR as just 28.1% expressed little knowledge about drug resistance (Supplementary Tables 1–4).

**Table 2:** Knowledge assessment regarding antibiotics and correct responses of the participants.

**Knowledge regarding the identification of antibiotics**	CR*n (%)
Have you ever heard of a type of medicine called antibiotics?	56 (58.3)
Is amoxicillin an antibiotic?	42 (43.8)
Is paracetamol an antibiotic?	51 (53.1)
Is aluminum hydroxide + magnesium hydroxide (antacid) an antibiotic?	38 (39.6)
**Knowledge regarding the uses of antibiotics**	
Are antibiotics useful for killing germs?	52 (54.2)
Are antibiotics often needed for cold and flu illnesses?	18 (18.8)
Does diarrhea get better faster with antibiotics?	59 (61.5)
Can antibiotics kill “good bacteria” present in our bodies?	48 (50)
Can antibiotics cause secondary infections after killing good bacteria present in our bodies?	46 (47.9)
Can antibiotics cause allergic reactions?	65 (67.7)
**Knowledge regarding ABR**	
If bacteria are resistant to antibiotics, it can be very difficult to treat the infections they cause.	27 (28.1)
Did you hear the term ABR?	26 (27.1)
Storage of unnecessary antibiotics is one of the reasons for ABR.	30 (31.3)
If bacteria are resistant to antibiotics, it can be very difficult to treat the infections they cause.	35 (36.5)
Many infections are becoming increasingly resistant to treatment by antibiotics.	34 (35.4)
Misuse of antibiotics can lead to ABR.	35 (35.5)
**Knowledge score**	**Median (IQR)**
Knowledge regarding identification of antibiotics	2 (1–3)
Knowledge regarding uses of antibiotics	4 (2–5)
Knowledge regarding ABR	1 (0–3)

*CR, Correct Responses.

The prototype that predicted strong antibiotic knowledge and AMR found multiple meaningful associations. Each level of education, from elementary school to graduation, was strongly linked to superior knowledge (*p* < 0.05). Knowledge also had a relationship with age group (*p* < 0.05), occupation (*p* < 0.05), and family income (*p* < 0.05), all of which have been proven to be favorable to the knowledge of antibiotics due to their level of education (**Table 5**). The type of family structure, the number of family members, and the type of infectious disease were not significant indicators of antibiotic use or knowledge of AMR (Supplementary Tables 1–4).

### HHs antibiotics knowledge

#### HHs antibiotics identification

The households were (*n* = 55, 57.3%) aware of the term “antibiotic” and that such medicines can fight against infectious diseases. Participants reported correct responses related to (*n* = 42, 43.8%) amoxicillin as an antibiotic, and more than half (*n* = 50, 52.1%) identified paracetamol as not an antibiotic (Supplementary Table 1). Knowledge related to the “other than antibiotics” category was recognized as antibiotics, and a very low number responded correctly (*n* = 38, 39.6%). The Median (IQR) Knowledge score regarding the identification of antibiotics was 2 (1–3) (Table 2).

#### HHs antibiotics use

Most of the HHs believed that antibiotics killed the germs (*n* = 52, 54.2%), and only half (*n* =48, 50%) knew “good bacteria” was present in everybody's bodies (Supplementary Table 2). Despite the frequent use of antibiotics, more than half of the HHs (*n* = 65, 67.7%) provided accurate information regarding allergic reactions as HHs experienced such allergic reactions. They reported numerous instances of adverse reactions caused by antibiotics.

### HHs knowledge related to ABR

The majority of the HHs (*n* = 70, 72.9%) had not heard the term ABR before and they showed less awareness and knowledge about ABR. As evident from their responses, many of them provided incorrect answers about the treatment after bacteria/germs get resistant to the antibiotics; 45.8% (*n* = 44) responded with “don't know” about such incidents (Supplementary Table 4).

Respondents believed that the storage of unnecessary antibiotics is not a cause for ABR and only 31.3% (n=30), answered correctly. The Median (IQR) Knowledge score regarding ABR was 1(0–3). A majority of the respondents also had misconceptions regarding the misuse of antibiotics and it was common among HHs (*n* = 35, 35.5%) (see Table 2).

### Overall understanding of antibiotics use, ABR, and storage of antibiotics in households

#### Likert scale responses

The Likert scale was used to measure the responses from HHs ranging from “strongly agree” to “strongly disagree.” Less than half of the participants (*n* = 33, 34.4%) agreed that antibiotics should be identified alongside other medications, even if they are prescribed separately. The urinary and respiratory tract infections can be treated only with antibiotics (*n* = 34, 35.4%) and just a small proportion of HHs had such knowledge. Only 25 HHs strongly agreed with the statement regarding penicillin-triggered allergic reactions and the test dose (*n* = 35, 36.5%). The majority of HHs (*n* = 50, 52.1%) were unconcerned about the statement that ABR is caused by antibiotics failing to kill or inhibit bacterial growth. Within households, family members shared antibiotics and less than half of HHs (*n* = 29, 30.2 %) agreed to the prospect of ABR owing to an incomplete antibiotic course. ABR can develop due to incomplete doses (as a result of HHs storing antibiotics for future illnesses), and the majority of HHs remained indifferent (*n* = 33, 34.4%), with fewer than half (*n* = 29, 30.2%) agreeing with the assertion on ABR (refer Table 3).

**Table 3:** Overall HHs knowledge related to antibiotics use, ABR, and infectious diseases.

**Questions**	LS*	***n* (%)**
I can recognize antibiotics in my prescription as I always	SA	14 (14.6)
differentiate antibiotics from other medicines.	AG	33 (34.4)
	NT	24 (25)
	DA	22 (22.9)
	SDA	3 (3.1)
LRTIs, URTIs, and UTIs are only treated by antibiotics.	SA	11 (11.5)
	AG	34 (35.4)
	NT	34 (35.4)
	DA	13 (13.5)
	SDA	4 (4.2)
Antibiotics such as penicillin can cause allergic reactions,	SA	25 (26)
if not checked with the patient with the test dose.	AG	35 (36.5)
	NT	24 (25)
	DA	10 (10.4)
	SDA	2 (2.1)
ABR is referred to the inability of antibiotics to	SA	23 (24)
kill/inhibit microorganisms' growth.	AG	22 (22.9)
	NT	50 (52.1)
	DA	1 (1)
	SDA	0 (0)
HH storage of antibiotics for future illnesses can lead to	SA	20 (20.8)
ABR.	AG	29 (30.2)
	NT	33 (34.4)
	DA	12 (12.5)
	SDA	2 (2.1)
Sharing of antibiotics in HH members can develop ABR.	SA	12 (12.5)
	AG	29 (30.2)
	NT	48 (50)
	DA	6 (6.3)
	SDA	1 (1)
Self-medication with antibiotics is one of the reasons for	SA	18 (18.8)
ABR.	AG	23 (24)
	NT	46 (47.9)
	DA	8 (8.3)
	SDA	1 (1)
Consultation with a physician for the (RTIs and UTIs)	SA	15 (15.6)
illness and pharmacist counseling can enhance the	AG	22 (22.9)
appropriate use of antibiotics.	NT	56 (58.3)
	DA	1 (1)
	SDA	2 (2.1)

^*^LS, Likert Scale (SA, strongly agree; AG, agree; NT, neutral; DA, disagree; and SDA, strongly disagree).

### HHs antibiotics storage

Antibiotics were stored at home by the majority of HH respondents (*n* = 80, 83.3 %) together with any other drugs for multiple purposes. Most of them were willing to show their stored antibiotics (*n* = 94, 97.9%), and a significant proportion of the antibiotics (*n* = 93, 96.9%) were in the oral dosage form. Antibiotics used for present illness by HHs (*n* = 50, 52.1%), were also used by other HH members (*n* = 46, 48.9%), and/or retained for future use. The antibiotics used by the majority of the HHs (*n* = 88, 91.7 %) for their current illness were purchased from the pharmacy/drug store staff (*n* = 41, 42.7 %). Antibiotics were available without prescription and only a small number (*n* = 23, 24%) of HHs got their antibiotics through prescription. Nearly half of the HHs (*n* = 47, 49%) received entire strips of antibiotics, more than the exact number required for the antibiotic course (refer to Table 4).

**Table 4:** HH storage of medications (antibiotics).

**Storage of medications**	***N* (%)**
**Do you currently keep antibiotics at home?**
Yes	80 (83.3)
No	16 (16.6)
**If yes, are you willing to show it/them to me?**
Yes	94 (97.9)
No	2 (2.1)
**Dosage form**
Oral	93 (96.9)
Other	3 (2.9)
**Status of antibiotics**
On use (first user)	50 (52.1)
On use (another person and for future use)	46 (48.9)
**Purpose of antibiotic use?**
For current illness	88 (91.7)
For future illness	3 (3.1)
Don't remember	3 (3.1)
For emergency use	2 (2.1)
**Who advised you to get the medicine?**
Physician	52 (54.2)
Pharmacy/drug outlet staff	41 (42.7)
Family/relatives/friends	3 (3.1)
**How have you got antibiotics?**
With prescription	73 (76)
Without prescription	23 (24)
**From where you have got antibiotics? (Source)**
Pharmacy	21 (21.9)
Drug store (small outlet)	75 (78.1)
**When/How long has it been?**
For the last 10 days	36 (37.5)
For last 20 days	35 (36.5)
From last month	17 (17.7)
More than 1 month	8 (8.3)
**Why was this source chosen?**
Easy access	75 (78.1)
Due to need	21 (21.9)
**Type of package?**
Blister pack	32 (33.3)
Strip pack	47 (49)
Bottle	15 (15.6)
Bottle and Strip	2 (2.1)
**Storage place**
Open place	5 (5.2)
Cabinet	6 (6.3)
Room	85 (88.5)
**Antibiotics name**
J01CA (penicillin)	11 (11.5)
J01CA04 (amoxicillin)	18 (18.7)
J01CR (amoxicillin + clavulanic acid)	30 (31.2)
J01FA10 (azithromycin)	38 (39.6)
J01FA09 (clarithromycin)	6 (6.3)
J01FA01(erythromycin)	6 (6.3)
J01AA07 (tetracycline)	8 (8.3)
J01AA02 (doxycycline)	13 (13.5)
J01MA02 (ciprofloxacin)	24 (25)
J01MA12 (levofloxacin)	12 (12.5)
J01MA01(ofloxacin)	13 (13.5)
J01DB05 (cefadroxil)	5 (5.2)
J01DD04 (ceftriaxone)	23 (24)
J01DD08 (cefixime)	12 (12.5)
J01FF01 (clindamycin)	2 (2.1)
J01XA01 (vancomycin)	6 (6.3)
J01XD01 (metronidazole)	31 (32.3)
**More than one antibiotic**
J01MA02 + J01XD01	19 (19.8)
J01CA04+ J01FA09 + J01MA12 + J01FA10 + J01XD01	14 (14.6)
**Number of unnecessary HH storage of antibiotics**	
1–2	62 (64.6)
3–4	29 (30.2)
5–6	5 (5.2)

#### Antibiotics at HHs

**Figure 1:**
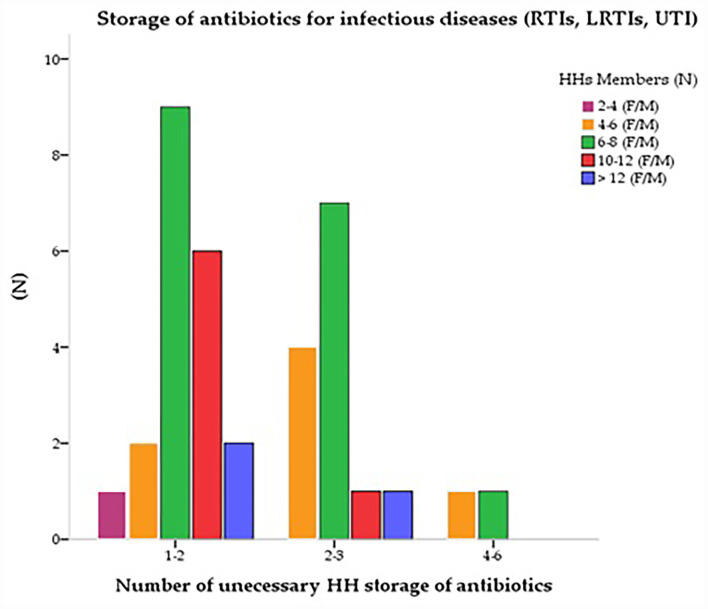
The total number of HHs vs. antibiotics storage for infectious diseases.

Azithromycin (J01FA10) was the most stored antibiotic (*n* = 38, 39.6%) under different brand names. The second most stored antibiotic was “amoxicillin along with clavulanic acid” (J01CR), a common brand of the multinational pharmaceutical industry (*n* = 30, 31.2%), and “amoxicillin” (J01CA04) (*n* = 18, 18.7%). The fluoroquinolones group “ciprofloxacin” (J01MA02) was found as the third most reported (*n* = 24, 25%) antibiotic among HHs. Third-generation cephalosporin “ceftriaxone” (J01DD04) which is common in rural areas was reported the fourth (n=23, 24%) most stored antibiotic among HHs, (*n* = 12, 12.5%) followed by “cefixime” (J01DD08). Few HHs also had “levofloxacin” (J01MA12) and ofloxacin (J01MA01) (*n* = 12, 12.5% and *n* = 13, 13.5%) respectively. Metronidazole (J01XD01) use was found concurrently (n=31, 32.3%) with other antibiotics to treat various infectious diseases. Many HHs stored more than one antibiotic in combinations. They had several unnecessary 1–2 (*n* = 62, 64.6%) and 3–4 (*n* = 29, 30.2%) antibiotics at their homes (Table 4). Figure 1 shows the storage of antibiotics at home for various infectious diseases based on the total number of its members. Except for two to four family members, who had a significant correlation with antibiotic storage (*p* < 0.05) and maintained fewer antibiotics at home than the other family members, the cross-tabulations reveal that the number of family members had no bearing on how antibiotics were stored (Figure 1).

### Comparison of HHs demographic characteristics and antibiotics uses and storage

Various demographic parameters were checked against antibiotics knowledge and use scores. Different age groups had a strong association (*p* = 0.017, H = 12) and affected the mean knowledge scores related to antibiotic use. Education was one of the main factors that determined proper storage and use, and this study found a significant association (p=0.001, H= 52.8) between education and HHs' understanding of antibiotics. Similarly, employment had a direct effect on proper antibiotic use and a strong association (*p* = 0.04, H = 10) was observed between employment and HHs' perspectives. The family setup (*p* > 0.05, H = 0.7), the total number of family members (*p* > 0.05, H = 0.5), and monthly income (*p* > 0.05, H = 7.3) of HHs did not affect the understanding of antibiotics identification, use, and ABR and no relationship was found among the variables. Although, family members having more than one chronic disease (*p* = 0.09, H = 0.8) had a near significant relationship in their understanding of antibiotics and their uses (*p* = 0.016, U = 735) (see Table 5).

**Table 5:** Comparison of demographic characteristics with respect to knowledge regarding the identification of antibiotics.

**Variables**	**Groups**	***N***	**Mean Rank**	**H**	*p*-value*
Age	18–28	45	50.42	12	0.017
	29–38	27	58.52		
	39–48	14	38.11		
	49–58	6	25.67		
	>60	4	29.88		
Education level of the participants	Primary education	28	32.77	52.8	0.001
	Secondary education	18	58.56		
	Higher secondary education	15	64.37		
	Graduation	13	80.96		
	Postgraduation	4	64.38		
	No formal education	18	22.72		
Occupation of the participants	Govt employee	19	61.42	10	0.04
	Private employee	23	45.15		
	Self-business	32	41.94		
	Daily wager	14	42.21		
	Other	8	64.69		
Family set-up of the participants	Individual	60	49.58	0.7	0.7
	Joint family	35	47.23		
	Extended family	1	28.5		
Total number of family (male and female)	2–4	4	55.75	0.5	0.9
	4–6	27	49.83		
	6–8	32	46.53		
	10–12	21	47.88		
	>12	12	49.42		
Average Monthly Income of the Participants (PKR)	1,000–20,000	9	56.11	7.3	0.1
	21,000–30,000	29	44.29		
	31,000–40,000	21	37.98		
	41,000–50,000	9	58		
	> 50,000	28	55.25		
Type of the infectious diseases	Lower respiratory tract infections	35	44.27	8	0.09
	Upper respiratory tract infections	11	35.05		
	Other infectious diseases (TB, etc.)	13	59.15		
	Chronic illnesses	29	55.72		
	Not any disease (NAD)	8	42		
Number of chronic diseases	**Groups**	**N**	**Mean Rank**	**U**	***p*** -value**
	0	33	57.73	735	0.016
	1–2	63	43.67		

p ≤ 0.05 was considered statistically significant. ^*^p-value was calculated using the Kruskal Wallis H test. ^**^p-value was calculated using the Mann Whitney U-test.

The overall results show that immediate attention is needed to enhance the rational use of antibiotics in households that do not have adequate knowledge of antibiotics, their identification, proper uses, and ABR-related factors.

## Discussion

The current study is the first of its kind in Pakistan's post-conflict districts. The results of this survey provide an up-to-date picture of HHs and medicines storage (antibiotics), which will contribute to the development of community education initiatives to promote sensible antibiotic use. The purpose of this study was to examine the knowledge and habits of HHs on antibiotic use and storage. Instructing HHs on medicines and their storage is one of the most important aspects of antibiotic use after receiving them from the community pharmacy, as the rest of the therapy outcomes are dependent on their proper intake at home. Our study showed a significant lack of awareness regarding the proper use of antibiotics, ABR, and needless medication storage at home, which indicated poor antibiotic practices at HHs.

The accurate identification of antibiotics is essential. Knowledge gaps in antibiotics use can lead to ABR, and we observed such a lack of knowledge among HHs in the present study. We found that antibiotic identification was relatively poor (39.6%) and similar results have been reported by Atif and his colleagues, and Tawseef et al., in their studies (34, 35). This problem is not limited to Pakistan alone; researchers from other countries have undertaken similar studies, particularly among rural low and low middle-income countries (LMIC) communities with varying levels of knowledge ranging from moderate to poor, touch comparisons are difficult (32, 36–38). As a result, our findings, as well as those of other research that looked into antibiotic knowledge in greater depth, show that there is still a lot of work to be done in terms of educating rural populations in LMICs about antibiotics and the proper use (31, 39, 40). Comparable results have also been reported in China where knowledge gaps and unfavorable attitudes regarding the proper use of antibiotics were detected among HHs. About 48.2% of respondents said they had used antibiotics for children without a prescription (41). Antibiotic knowledge is also low in countries in the Middle East, especially in Saudi Arabia; according to a survey, a majority of respondents sought antibiotics without a prescription (42). According to the reported study, 71% of Saudis and 36% of Kuwaitis did not complete their antibiotic medication course (42, 43). Findings from Jordan were similar to our results, with 60.7% of individuals having an insufficient understanding of the antimicrobial resistance (44). Compared to its neighboring countries, antibiotic misuse in Qatar is extremely high (85%) (45). Patients took antibiotics without knowing it and were unfamiliar with the terms, particularly “ABR” and comparable findings have been reported from other countries as well.

One of the most important findings of the current study is that respondents incorrectly believed that they could stop taking antibiotics midway without completing the course; about 43% of the Serbian population also believes likewise (46). The situation is particularly bad in African regions, where an urgent call for work on an action plan against ABR is required to address the problem. Equally, India, Iran, and other Asian countries also need extensive attention (47–49). According to a comparable study from South Africa, a better level of education will boost antibiotic awareness and proper use among consumers (50, 51). To avoid ABR and inappropriate use, antibiotic dosing regimens must be finished within the timeframe given (five to seven days or as recommended). Summarizing the current situation in European countries A Machowska and C S Lundborg highlight that major factors driving ABR among the general public are lack of public knowledge and awareness, access to antibiotics without prescription, and leftover antibiotics at home (52). According to the latest Eurobarometer survey, 34% of Europeans used antibiotics at least once in 2016 (52). Antimicrobial/antibacterial resistance is not only an issue in developing countries but also a major problem in developed countries. All governments and regions must investigate the irrational use of antimicrobials to establish effective strategies to combat antimicrobial resistance (51, 53). Our results could be related to those from other low and middle-income countries (38), as well as Germany, the United Kingdom, Sweden, Italy, Poland, Lithuania, Cyprus, Siberia, and Hong Kong. The present finding emphasized the appropriate use of antibiotics among HHs and discourage the unnecessary storage of antibiotics through patients' education and awareness in the community.

In the current study, the majority of customers preferred to store unnecessary antibiotics for future usage, and near similar findings have been reported from China (31%) and Jordan (49%) (44, 54). The most common way to access nonprescription antibiotics is through leftover medicines. Non-prescription antibiotic use among children in HHs was found to be significantly linked to antibiotic storage at home (41). Moreover, more than half of the total HHs in this study were willing to keep antibiotics at home, which is a little different from studies in Indonesia, Iran, Iraq, Oman, Greece, and the United States, where 82 to 100% percent of HHs stored antibiotics at home (17). When it came to keeping antibiotics or other medicines, families with members who had chronic illnesses were more inclined to do so than those who did not experience such illness. The likelihood of antibiotics storage was also associated with HHs occupation. Other research studies have come to similar conclusions (2, 17, 55). Antibiotics that have been left over should be disposed of correctly. Other countries' experiences can be beneficial to Pakistan. Residents of Portugal, Sweden, and the Netherlands, for example, regularly return unused antibiotics to the pharmacy (30, 56). The Starfish Project in the United States gathers unwanted antibiotics from HHs through the use of free mail labels (30). While a lack of public awareness about inappropriate antibiotic use in self-limiting diseases contributes to the high prevalence of over-the-counter antibiotic purchases, easy access to over-the-counter antibiotics is also a factor in leftover antibiotics as reported in similar other studies (30, 57). According to Mutaseim Makki and his colleagues, the incidence of unused prescriptions in HHs has grown drastically in recent decades, resulting in medicines wastage (58). Patients' non-adherence to medication-taking was shown to account for 50% of unnecessary storage which leads to wastage (58).

According to studies, areas that have experienced continuous conflict are prime locations for bacteria to develop resistance and misuse of antibiotics (59). Violent conflict and displacement heighten the difficulties of limiting the emergence and spread of AMR. LMICs affected by conflict must not only deal with resource constraints but also discover ways to care for and treat injured people carrying more resistant infections (60). According to the literature and expert opinions, better lab diagnoses, the establishment of surveillance systems, and infection prevention and control should be prioritized along with AMR control strategies in the conflict-affected LMICs (61). However, there is a lack of research on the usefulness of these strategies in such conflict situations. More research studies are needed in post-war zones so that effective treatments can be devised and implemented.

Public awareness efforts and awareness campaigns are needed to discourage people from using leftover antibiotics at home for themselves or their families and to encourage people to properly dispose of leftover medicines. Our findings show that personalized interventions can reach people with a wide range of demographic and socioeconomic factors. Family members with a history of infectious diseases like respiratory tract infections were a significant risk factor for keeping antibiotics at home, according to our findings (43, 44).

“World Antibiotic Awareness Week” was organized by the National Institute of Health (NIH), Pakistan, on 18–24 November 2021. Such occasions can result in a step-up in Pakistani society's consciousness. The NIH must stress the significance of not keeping antibiotics at home and educate HHs in the affected areas. Therefore, now is an excellent time (spring) to launch a program for HHs and communities, particularly in Pakistan's post-conflict rural areas, to inform the people about the proper use of antibiotics and the adverse effects (ABR) of inappropriate antibiotics consumption. The findings revealed by Khan et al. (57), are very comparable to the current research conducted in post-conflict regions (5). The consumer-related factors were evaluated at community pharmacies through quantitative and qualitative approaches (38).

There are a few limitations that should be considered while interpreting the results of our study. First, it was a cross-sectional study of Pakistan's post-conflict areas that focused on the most impacted districts from militancy and army operations. It did not cover the rest of the Khyber Pakhtunkhwa, Malakand division districts; thus, the findings cannot be generalized. However, these results can be considered for similar other conflict areas in the country. Second, due to cultural norms, women's participation was not documented, as the men always represent their families during any visit to the HHs in these areas; all these limitations are an integral part of the rural areas of Swat, Khyber Pakhtunkhwa customs, and traditions. However, we believe that female participation will not largely impact the results as these regions are primarily led by men who are decision-makers in their families and a convenient sampling method was employed. Third, the sample was taken from only two sites and the sample size is small. Fourth, we only included 'antibiotics' among the stored medications at home, no additional medications were stated by the HHs. In Pakistan's rural areas, more research on medicines storage among HHs is required.

## Conclusion

This study showed that storage of antibiotics for future use is quite common among HHs residing in post-conflict regions of Pakistan. Households identified antibiotics among the stored drugs based on packaging and several common brands but were unable to explain why leftover antibiotics should be saved for future use as the storage of antibiotics was observed. The majority of HHs were not aware of ABR and did not explain how antibiotics cause resistance. Furthermore, ABR is the result of irrational antibiotic use, and antimicrobial/antibiotic oversight measures are still weak in Pakistan. The ABR and antibiotic stewardship are still unknown to the rural population. In terms of healthcare, Pakistan's post-conflict areas are the most neglected. More research studies are needed to raise awareness of the ABR problem and to educate HHs on how to use antibiotics properly when they are needed.

## Data availability statement

The raw data supporting the conclusions of this article will be made available by the authors, without undue reservation.

## Ethics statement

The studies involving human participants were reviewed and the Ethics Committee for Medical Research at Xi'an Jiaotong University has given its approval to our study (Ref.No: 2020-1342). The patients/participants provided their written informed consent to participate in this study.

## Author contributions

FaiK and YF: the study concept and design. FaiK: writing of the manuscript. QK: data analysis. TA and FarK: obtaining data. TM and KH: interpretation of results. TM and YK: critical revisions of the manuscript. All authors approved of the version for submission.

## Funding

This work was funded by the National Natural Science Fund (71974156), Young Talent Support Plan, High Achiever Plan of Health Science Center, Xi'an Jiaotong University, and the Central University Basic Research Fund (2015qngz05).

## Conflict of interest

The authors declare that the research was conducted in the absence of any commercial or financial relationships that could be construed as a potential conflict of interest.

## Publisher's note

All claims expressed in this article are solely those of the authors and do not necessarily represent those of their affiliated organizations, or those of the publisher, the editors and the reviewers. Any product that may be evaluated in this article, or claim that may be made by its manufacturer, is not guaranteed or endorsed by the publisher.
