# Supplementary material for: Assessment of antibiotic storage practices, knowledge, and awareness related to antibiotic uses and antibiotic resistance among household members in post-conflict areas of Pakistan: Bi-central study
Source: Front Med (Lausanne). 2022 Sep 8;9:962657. doi: 10.3389/fmed.2022.962657 (PMC9494294; doi:10.3389/fmed.2022.962657)
Supplement: Supplementary file 1 [file Data_Sheet_1.PDF]

Supplementary Tables (1-4)

Table 1: Factors related to identification of antibiotics

**Factors related to identification of antibiotics**	**n (%)**
**Have you ever heard of a type of medicine called antibiotics?**	
Yes	55 (57.3)
No	27 (28.1)
Do not know	14 (14.6)
**Is amoxicillin an antibiotic?**	
Yes	42 (43.8)
No	10 (10.4)
Do not know	44 (45.8)
**Is paracetamol an antibiotic?**	
Yes	18 (18.8)
No	50 (52.1)
Do not know	28 (29.2)
**Is aluminum hydroxide + magnesium hydroxide (antacid) an antibiotic?**	
Yes	15 (15.6)
No	39 (40.6)
Do not know	42 (43.8)

Table 2: Knowledge regarding use of antibiotics

**Knowledge regarding use of antibiotics**	**n (%)**
**Are antibiotics useful for killing germs?**	
Yes	52 (54.2)
No	13 (13.5)
Do not know	31 (32.3)
**Are antibiotics often needed for cold and flu illnesses?**	
Yes	59 (61.5)
No	18 (18.8)
Do not know	19 (19.8)
**Does diarrhea get better faster with antibiotics?**	
Yes	59 (61.5)
No	10 (10.4)
Do not know	27 (28.1)
**Can antibiotics kill "good bacteria" present in our bodies?**	
Yes	48 (50)
No	15 (15.6)
Do not know	33 (34.4)
**Can antibiotics cause secondary infections after killing good bacteria present in our bodies?**	
Yes	46 (47.9)
No	11 (11.5)
Do not know	39 (40.6)
**Can antibiotics cause allergic reactions?**	
Yes	65 (67.7)
No	13 (13.5)
Do not know	18 (18.8)

Table 3: Knowledge regarding antibiotic resistance

**Knowledge regarding antibiotic resistance**	**n (%)**
**If bacteria are resistant to antibiotics, it can be very difficult to treat the infections they cause.**	
Yes	27 (28.1)
No	9 (9.4)
Do not know	60 (62.5)
**Did you hear the term antibiotic resistance?**	
Yes	27 (28.1)
No	22 (22.9)
Do not know	47 (49)
**Storage of unnecessary antibiotics is one of the reasons for antibiotic resistance.**	
Yes	30 (31.3)
No	5 (5.2)
Do not know	61 (63.5)
**If bacteria are resistant to antibiotics, it can be very difficult to treat the infections they cause.**	
Yes	35 (36.5)
No	12 (12.5)
Do not know	49 (51)
**Many infections are becoming increasingly resistant to treatment by antibiotics.**	
Yes	34 (35.4)
No	18 (18.8)
Do not know	44 (45.8)
**Misuse of antibiotics can lead to antibiotic resistance.**	
Yes	35 (36.5)
No	14 (14.6)
Do not know	47 (49)

Table 4: Knowledge regarding antibiotic resistance

**Knowledge regarding antibiotic resistance**	**n (%)**
If bacteria are resistant to antibiotics, it can be very difficult to treat the infections they cause.	
Yes	27 (28.1)
No	9 (9.4)
Do not know	60 (62.5)
Did you hear the term antibiotic resistance?	
Yes	27 (28.1)
No	22 (22.9)
Do not know	47 (49)
Storage of unnecessary antibiotics is one of the reasons for antibiotic resistance.	
Yes	30 (31.3)
No	5 (5.2)
Do not know	61 (63.5)
If bacteria are resistant to antibiotics, it can be very difficult to treat the infections they cause.	
Yes	35 (36.5)
No	12 (12.5)
Do not know	49 (51)
Many infections are becoming increasingly resistant to treatment by antibiotics.	
Yes	34 (35.4)
No	18 (18.8)
Do not know	44 (45.8)
Misuse of antibiotics can lead to antibiotic resistance.	
Yes	35 (36.5)
No	14 (14.6)
Do not know	47 (49)
