# Supplementary material for: Assessment of antibiotic storage practices, knowledge, and awareness related to antibiotic uses and antibiotic resistance among household members in post-conflict areas of Pakistan: Bi-central study
Source: Front Med (Lausanne). 2022 Sep 8;9:962657. doi: 10.3389/fmed.2022.962657 (PMC9494294; doi:10.3389/fmed.2022.962657)
Supplement: Supplementary file 2 [file Data_Sheet_2.PDF]

BASELINE STUDY BOOKLET

Booklet Contents:
1.Information Sheets for the Participants
2.Informed Consent
3.Questionnaire

I. INFORMATION SHEET FOR THE PARTICIPANTS

Purpose of study:

You are invited to participate in a research study designed to determine the "**Households Knowledge towards Use and Storage of Antibiotics:**

By this study, we would like to draw the attention of members of society, government and non-governmental organizations, media, and other people working on grass root level towards the importance of rational use of antibiotics.

If you want to participate in the study, you will be interviewed by the data collector. All information collected from your participation will be confidential and it will be used only for the research purpose. This information will be destroyed at the end of the study. Your name will be kept confidential and will not be mentioned in any report or publication. Remember, this is not a test and there are no right or wrong answers.

II. INFORMED CONSENT

You will be asked to sign an informed consent stating that you understand the nature of the study and what is required from you in the study.

Participation in this study is voluntarily and will not affect your privacy. You are free at any time to change your mind and withdraw from the study without needing to justify your decision.

1. I, undersigned voluntarily, agree to take part in this study which I understand has been approved by responsible authorities.
2. I, confirm that a full explanation of the purpose and nature of the study has been explained to me.
3. I have been given the opportunity to ask questions on all aspects of the study and have understood the advice and information given as a result.
4. I agree to co-operate faithfully with the studying investigators with regard to my eligibility to participate in the study.
5. I also understand that failure to take part will no way prejudice my privacy.
6. I consent to the investigators having access to the information in my records, with the understanding that any publication shall not reveal my name or any other personal identifiers.

Signature: ____ **Date:** __________
(Volunteer)

I confirm that I have explained the nature and purpose of the study to this volunteer. If, at any time during the course of this study, new information develops that may affect the volunteer's willingness to continue participation, a statement of this information will be provided to him.

Signature: ____ **Date:** __________
(Researcher)

Interview with Household Member:

Household Code: ______________ **Interview Date:** _________________

Municipality/Village Development Committee: ________________________ **Union Council:** _____

Section 1: Socio-Demographic

Code	Name of Respondent	Relation to Head of Household	Age	Sex	Education
KK-01					

Note:

Sex: 1. Male 2. Female

Education: 1. Illiterate, 2. Literate, 3. Primary/Secondary, 4. High School, 5. Intermediate, 6. Bachelors, 7. Masters, 8. PhD

KK-02	What is your occupation, that is, what kind of work do you mainly do?	…………………………………
KK-03	What is your type of family?	Individual………...……………………1<br>Joint…………….…………..……..2<br>Extended……………………….…3
KK-04	How many members are in your family?	Total……………<br>Male……………<br>Female ………..
KK-05	What is your average monthly family income?	Salary: PKR…………………..<br>Agriculture: PKR……………<br>Business: PKR……………………<br>Others (Specify…………….): PKR………<br>Don't Know ……………………….
	Family members with chronic illness?<br>Total Patients with chronic illness____	Name of the Disease:<br>1.<br>2.<br>3.<br>4.
	Presence of a health professional in the household?<br>If yes, please Specify________________________	

Section 2: Knowledge

Code	Questions	Coding Category
KK-06	Have you ever heard of a type of medicine called antibiotics?	Yes…………………………….......1<br>No …..………………………….....2

Note: If respondent says 'No" please ask if they have heard of a widely used antibiotic such as penicillin or metronidazole before asking the questions from 107. (To show packs of the common antibiotics for recognition)

Intervention: The lay man should know after the interview; what's means by bacteria, antibiotics and resistance from antibiotics in very common in easy language

Codes	Domains	Questions	Response				
			Strongly Disagree	Disagree	Uncertain	Agree	Strongly Agree
KK-07	Identification of antibiotics	Amoxicillin is an antibiotic					
		Paracetamol is an antibiotic					
		Aluminium hydroxide+ Magnesium hydroxide (antacid) is an antibiotic					
KK-08	Knowledge on the role antibiotic	Antibiotics are useful for killing germs					
		Antibiotics are often needed for cold and flu illness					
		Diarrhea gets better faster with antibiotics					
KK-09	Side-effects of antibiotics	Antibiotics can kill "good bacteria" present in our bodies					
		Antibiotics can cause secondary infections after killing good bacteria present in our bodies					
		Antibiotics can cause allergic reactions					
KK-10	Antibiotic resistance	If bacteria are resistant to antibiotics, it can be very difficult to treat the infections they cause					
		Many infections are becoming increasingly resistant to treatment by antibiotics					
		Misuse of antibiotics can lead to antibiotic resistance					

3. Current medicine use and storage

Do you currently keep medicines at home?

Yes ☐ No ☐

If yes, are you willing to show it/them to me?

Yes ☐ No ☐

Medicine	Medication name, strength, dosage form	Current status of medicine "√"				Purpose of use (illness)
		On use		Kept for future use	Not for use	
		Original /first user	Another person			

Medicine number	Who advised to get the medicine?	How was it acquired? "√"		From where? (Source)	When/How long has it been?	Why was this source chosen?
		With Prescription	Without prescription			

Medicine number	Type of package		Packaging Material *	Expiry date "√"			Storage place
	Original	Other		Recorded		Not recorded	
				Expired	Not expired		

*Type of package: A: Plastic bag , B: Paper bag C: Blister pack D: Strip pack E: Bottle F: Vial G: Other (specify)

Thank you!
